# Conceptualising and addressing mental disorders amongst Muslim communities: Approaches from the Islamic Golden Age

**DOI:** 10.1177/1363461520962603

**Published:** 2020-10-15

**Authors:** Karim Mitha

**Affiliations:** 1 University of Glasgow, UK; 2 University of Edinburgh, UK; 3 Imperial College NHS Healthcare Trust, London, UK

**Keywords:** history of psychiatry, history of psychology, hospitals, Islamic medicine, madness, Muslim mental health

## Abstract

Although Islam is the world’s second-largest religion, there continues to be misconceptions and an overall lack of awareness regarding the religious and social worlds that make up the global Muslim community. This is particularly concerning when examining notions of mental ill-health, where a lack of cultural awareness, understanding, and sensitivity can impede adequate treatment. As a global religion, Islam is practiced within various cultural milieus, and, given the centrality of faith amongst Muslim communities, a conflation of religion and culture can occur when attempting to understand mental health paradigms. Whilst much of the discourse regarding Muslim mental health centres on cultural formulations, this article discusses how, historically, conceptualisations relating to medicine and mental health were ensconced within the particular medical paradigm of the day. Specifically, it considers the frameworks within which mental health and illness were understood within the medieval Muslim medical tradition and their relevance to contemporary debates in psychology and psychiatry. In sum, this paper seeks to demonstrate that cultural formulations of mental illness, often viewed as “Islamic”, are distinct from historical Islamic approaches to mental health which employed contemporaneous medical discourse and which act as the reference marker for the emergent revivalist Islamic psychology movement seen today.

## Introduction

There has been increased attention to mental health and illness in the lay public discourse, particularly in the last decade (Stickley, 2019). Concurrently, increasingly diverse populations in Euro-American contexts have called attention to the need for cultural competency and an awareness of cultural approaches to mental health and illness within ethnic minority communities. This is important because whilst there is increased awareness and willingness for people to seek help for mental health difficulties, the question of whether extant services are meeting the needs of the population remains open (Stickley, 2019).

Today, the dominant model of mental health utilised by clinicians globally is the biopsychosocial model (Patel, 2014). Nevertheless, this framework is not necessarily universally accepted and there is recognition that patients often have their own lay models of understanding. Alternative approaches to what is labelled “mental illness” include: popular and lay conceptions of “madness” (Scull, 2015; LaFrancois, Menzies, & Reume, 2013; Beresford & Russo, 2016); critiques from critical psychiatrists (Moncrieff & Cohen, 2009; Kirsch, 2015), cultural relativist models of distress (Lipsedge & Littlewood, 2005; Esmail, 1996), critical models that resist the pathologising of symptomatic responses to socio-environmental stressors, such as inequalities and austerity (Knight & Thomas, 2019), and models grounded in lay discourses of mental illness, which often explain such experiences within paradigms of demonic or spirit possession (Littlewood & Dein, 2013a; Dein, Alexander & Napier, 2008). Whilst cultural frameworks of minority ethnic patients tend to be “Other”-ed, it is important to note that, within Western contexts, the bio-medical model is itself a recent phenomenon and that, historically, those who would now be labelled as “mentally ill” were often considered to be possessed by demons and spirits (Dols, 1992; Mestiri, 2006; Rahman, 1989; Shoshan, 2003; Stanwell-Smith, 2019). This view was the most common framework used to understand mental ill-health in Medieval Europe (Porter, 1999), and indeed, arguably, still underlies contemporary lay perceptions of mental ill-health amongst many Muslim communities. However, it is worth noting that, historically, the Islamic world had quite different approaches towards mental health and illness. The Islamic perspective on mental health was a holistic one, in which positive mental health and good physical health were viewed as being interconnected. This is of religious importance, as Awaad and colleagues note: “the ‘preservation of intellect/mental capacity’ is regarded as one of the five major objectives of Islamic legislation in general (the other four are preservation of religion/faith, life, family, and wealth)” (Awaad, Mohammed, Elzamzamy, Fereydooni, & Gamar, 2019, p. 4).

Rahman (1989) explains that the Arabic word for “self”, *nafs*, can also be translated as “soul”, suggesting that Islamic doctrine views the concepts of self and soul as interlinked. This holistic view of a person follows from the Islamic conceptualisation of the symbiotic relationship between the body (*jism*) and spirit (*ruh*) (Ally & Laher, 2008; Deuraseh & Talib, 2005; Saniotis, 2018). Within Islamic doctrine, supplication to God must evoke physical and spiritual purity, and, as such, the tradition has religious obligations regarding sanitation and nutrition (e.g., *wudu, halal/haraam/makruh* foods). It has been argued that positive physical and mental wellbeing are essential for the performance of religious obligations such as prayer (Awaad, et al., 2019). Keshavarzi and Ali (2019) elaborate on this point about mental wellbeing, stating that, in the Islamic tradition, legal competence (*ahliyyah kamilah*) is ensured by the soundness of both physical and mental capacities. As a result, positive mental health and good physical health are interconnected and are both seen as necessary for performing one’s religious obligations (Awaad et al., 2019).

Within Muslim communities today, current lay models of mental ill-health appear largely to be based on cultural, pre-Islamic influences. For instance, there is the perception that mental illnesses may be caused by divine retribution, “evil eye” (*’ayn*), whisperings (*wiswaas*), magic (*sihr*), envy (*hasad*), or supernatural spirits, such as djinns (Carnevali & Masillo, 2008; Laird, de Marrais, & Barnes, 2007; Littlewood & Dein, 2013b; Ineichen, 2012; Haque, 2004; Islam & Campbell, 2014; Lim, Hoek, Ghane, Deen & Blom, 2018). These issues may also be viewed within a religious paradigm, such as djinn possession, lack of faith or not being a “good Muslim”, or a failure to pray. Treatment, in turn, can be based on reading of religious texts (i.e. the Qur’an) on a daily basis, performing *dhikr* (recitation or repetition of the names and attributes of God)*, ruqyah* (incantations for the purposes of exorcism), fasting, etc. (Bhugra, 1996; Beliappa, 1991; Cinnirella & Loewenthal, 1999; Abdel-Khalek, 2007; Weatherhead & Daiches, 2010; McClelland, Khanam, & Furnham, 2014; Mitha, 2019; Dein, et al., 2008; Pratt et al., 2016; Sheikh, 2005; Lim et al., 2018). The systematic review by Walpole and colleagues (2012) has suggested that, although there is a common perception that Muslims believe mental illness may be caused by divine retribution, “evil eye”, or supernatural spirits, the evidence base on Muslim mental health is lacking and religious and cultural views are often conflated by researchers, as well as by Muslims themselves. Keshavarzi and Haque (2013) noted that lay Muslims may be unaware of the distinction between the cultural and religious paradigms of mental illness. This supports the findings of Hussain and Cochrane (2002), who noted that their respondents turned to religious and cultural explanations if a clinical diagnosis did not accord with their personal worldview. They also stated that their respondents generally refuted cultural “healers”, as they were believed to be outwith the Islamic tradition, but seemed more receptive if those “healers” employed Islamic terminology, thereby rendering them more religiously permissible (Hussain & Cochrane, 2002). This has been observed across various Muslim societies. Razali and Tahir (2017), for instance, found that amongst Muslims in Malaysia, local practises termed “Islamic medicine” were local cultural practises labelled with Islamic terminology. On the other hand, Mir and colleagues (2015) have incorporated religious frameworks into bio-medical based interventions for depression, making use of religious narratives in behavioural activation therapies, following the approach of religiously-based cognitive behavioural therapy (Adewoye, 2016; Mir et al., 2015).

The movements of “Muslim mental health” and “Islamic psychology” have emerged, arguably, as a response, with intra-community organisations in the United Kingdom and the United States marketing courses on “Islamic psychology”. However, as al-Karam (2018) notes, the exact scope and entailments of “Islamic psychology” remain open to debate. Younis (2019) notes that whilst its broad aims are to address stigma and develop cultural competency for clinicians working with Muslim populations, he also notes its emergence within a sociological movement of “revivalist Islam”, with the “Islam-icising” of terms, essentially creating a “market” of “Muslim mental health”. This leads one to ask: does the Muslim mental health movement just amount to “Islam-icising” cultural terms, be they Western or Eastern? Is it mainly about cultural competency and stigma? Or does it instead involve the development a distinct paradigm for mental health, based on an understanding of psychological principles within a religious framework? This article argues for the latter view: namely, that distinct Islamic approaches to mental health were historically developed, alongside developments in the fields of medicine and public health during the Islamic Golden Age.

During the Islamic Golden Age, roughly corresponding to the 8th to 15th centuries of the Gregorian calendar, Islamic culture and civilisation thrived and cultivated new developments in the arts and sciences. This period has often been of interest to medical historians (see, e.g., Browne, 1921; Carnevali & Masillo, 2008; Dols, 1984, 1992; Ullmann, 1978; Pormann & Savage-Smith, 2007; Porter, 1999; Shoshan, 2003) and contemporary Muslim scholars (e.g., Ammar, 1984; Awaad & Ali, 2015; Badri, 2013; Hamarneh, 1983; Khan, 1986; Mestiri, 2006; Omrani, Hotlzman, Akiskal & Ghaemi, 2012), including most recently Awaad and colleagues (2019). These scholars have sought to advance our understanding of this time period in critiquing the idea that Muslim scholars were simply translators and “holders” of Hellenestic theories until Europe was able to “reclaim” them during the Renaissance. Additionally, while they report broadly similar findings about historical events, these scholars have sought to examine the events of the Islamic Golden Age through the particular lenses of their disciplinary specialities and thus have focused on slightly different areas of significance.

Whilst Europe was in the Middle Ages, Muslim civilisations prospered and their achievements in the transmission of knowledge, the development of public institutions, and translation aided in the advancement in science, mathematics, and medicine. Public health initiatives, such as hospitals (*bimaristans*), emerged, as did centres for medical education (Ammar, 1984; Dols, 1984; Hamarneh, 1983; Mestiri, 2006; Pormann & Savage-Smith, 2007; Rahman, 1989). These extended into models of thinking about health and illness, including what we would now conceptualise as mental illness. Contemporary scholarship in Muslim mental health and Islamic psychology has often looked to this period to examine Islamic concepts of mental health and illness. As previously mentioned, the emergence within Britain and America of intra-community courses on “Islamic psychology” claiming to use or to be informed by scholarship from the Islamic Golden Age demonstrates the need for a critical examination of approaches to mental health from this period, and additionally demonstrates the importance of increased scholarship about Muslim approaches to mental health as an emerging field of study.

## Conceptualisations of health and illness in the Islamic Golden Age

Pre-Islamic Arabic medicine was replete with discourse about supernatural phenomena, such as magic, evil eye, talismans and charms, shamans, spirits, etc. (Hamarneh, 1983; Dols, 1992; Mestiri, 2006; Ullmann, 1978). These beliefs were passed on through the generations as folk models of health and illness. With the advent of Islam, these paranormal practises largely fell by the wayside as the Prophet Muhammad forbade the use of black magic (Awaad et al., 2019; Dols, 1992; Mestiri, 2006; Porter, 1999; Shanks & al-Kalai, 1984; Ammar, 1984; Khan, 1986). It was through the Islamic tradition of acquiring knowledge that scholars during the Islamic Golden Age expanded on existing medical models from the Hellenestic tradition, such as from Hippocrates, Galen, and the humoural theory of medicine. This is distinct from what is known as Prophetic medicine (*al-tibb al-nabawi*), which refers to treatments attributed to the Prophet in the Hadith (traditions and sayings attributed to the Prophet Muhammad) (Ullmann, 1978). The use of religious and medical forms of healing co-occurred – for instance, the use of prayer and ritual healing in addition to using treatments according to the medical model of the time (Dols, 1992).

As Islam spread, Muslims came into contact with other civilisations and new ways of thinking. Islamic science became influenced by existing schools of thought in India, Persia, and Greece (Ammar, 1984; Hamarneh, 1983; Mestiri, 2006; Pormann and Savage-Smith, 2007; Ullmann, 1978). Those views that were seen by scholars as not contradicting Islamic ethics and tradition became assimilated and disseminated (Hamarneh, 1983). Works from these cultures were translated into Arabic, which became the *lingua franca* of science and medicine during that time (Ammar, 1984; Dols, 1984; Ullmann, 1978; Watt, 1972). With the greater spread of these materials in Arabic, Islamic medicine began to emerge drawing on the prevailing framework of the time - that of Galen and the humoural theory of medicine. This theory postulated that the body is comprised of four humours: black bile, yellow bile, blood, and phlegm (Browne, 1921; Dols, 1984; Hamarneh, 1983; Mestiri, 2006; Pormann & Savage-Smith, 2007). These humours correspond to the four elements of: earth, fire, air, and water; and the four qualities of: dry-cold, dry-hot, moist-warm, and wet-cold (Dols, 1984; Pormann & Savage-Smith, 2007; Awaad & Ali, 2015). To be in proper health, it was believed that the four humours ought to be in equilibrium, and, subsequently, poor health was due to an imbalance (Dols, 1984; Pormann & Savage-Smith, 2007).

This framework also existed in Europe in the Middle Ages. However, there was no expansion of Galenic and Hippocratic concepts in Europe, in contrast to the Islamic world and the development of Islamic medicine and approaches to mental health (Biller & Ziegler, 2001). Instead, the lay population relied mostly on folk healers, superstitions, and religious practises as forms of treatment. In Medieval Europe, it was believed that illness was due to sin (drawing on the concept and doctrine of “Original Sin”, which does not exist in the Islamic tradition) or demonic possession, that suffering was needed for Salvation, and that God controlled any healing – thus distinguishing medical knowledge from religious practice (Biller & Ziegler, 2001; Perez, Baldessarini, Undurraga, & Sanchez-Moreno, 2012; Wallis, 2010). Additionally, the visitation of shrines of saints or astrologers was also a healing practice in Europe (Bovey, 2015). Wallis (2010) notes a distinction between *medicina*, as theory and practice, versus *physica*, formalised study and teaching through texts, and argues that the former dominated Medieval European practice. Thus, according to Wallis (2010), although there was an awareness of *medicina*, it was seen as a more secular entity and distinct from religious knowledge. Wallis (2010) further elaborates that formalised and systematic approaches towards practising and educating practitioners, or *physica,* did not occur in Europe until the late Middle Ages. This stands in contrast with the development of more professionalised forms of medical education and systematic approaches to medical practice that occurred during the Islamic Golden Age, as will be discussed later in this article.

In contrast to the approaches in Medieval Europe, scholars in Muslim lands adapted, refined, and expanded on the Galenic medical models. Abu Zayd Hunayn ibn Ishaq al-‘Ibadi (809-874) was perhaps one of the first physicians in Muslim lands to extend Galen’s ideas and subsequently influenced future Muslim physicians in the Golden Age. In his treatise, *Masa’il fil Tibb lil-Muta’alimin*, he said that there were seven natural matters: the elements (fire, air, water, earth), the body’s temperature, the humours, major and minor organs, natural powers, actions and reactions, and spirits (natural, animal, psychic). He said that the six non-naturals (circumstances that people themselves could control and natural processes within the body) were: air, regular intake of food and drink, work (including physical exercise) and rest, wakefulness and slumber, vomiting and the use of enemas, and whatever affects us emotionally, such as worry, fear, anger, and joy (Hamarneh, 1983; Khan, 1986; Pormann & Savage-Smith, 2007). Many of the works by Muslim physicians, especially by Ibn Sina (930-1037) and Abu Bakr Muhammad bin Zakariyyah al-Razi (865-925), were not only influential for their times in describing various illnesses, but also for discussing and re-formulating views on mental health. It is to this that we now turn.

## Notions of madness

Scholars during the Islamic Golden Age understood that there were conditions which affected one’s psychological and spiritual states. Dols (1992) uses the term *majnun* (madness) to refer to those states of mind that scholars viewed as being the consequence of an imbalance of these psychological and spiritual states. Shoshan (2003), however, takes issue with this terminology, arguing that the *majnun* that Dols (1992) describes may actually refer to a culturally determined label of madness, and that it is not indicative of current contemporary conceptualisations of mental illness. Deuraseh and Talib (2005) and Saniotis (2018) suggest the appropriate terms for the conceptualisation of mental health would be that of *al-Tibb al–Ruhani* (spiritual/psychological medicine) and *al-Tibb al-Qalbi* (mental medicine), as used by scholars/physicians of the era. According to al-Ghazali (1058-1111), the renowned philosopher, the spiritual nature, or soul, consists of the heart (*qalb*), spirit (*ruh*), intellect (*‘aql*), and self (*nafs*). He argued that an interconnectedness and balance between them is essential for spiritual identity and thus maintaining connectivity to God and that deviation from this results in abnormality (Haque, 2004; Keshavarzi & Haque, 2013; Mohamed, 1986). Some scholars have attempted to overlay the Islamic conceptualisation of the soul, *nafs*, onto the Freudian psychoanalytical approaches to the “self” – where *al-nafs al-ammara* is seen as the lowest element, at mercy to animalistic temptation and inclination (i.e., the id), *al-nafs al-lawwama* which enables reason and decision making and self-reflection (i.e., the super-ego), and *al-nafs al-mutmaenna* corresponding to inner peace, tranquility, satisfaction, and self-actualisation and the desired state of attainment, which has been linked to the concept ‘*aql* (reason/intellect). However, the appropriateness of this like for like typology been also called to question (Mitha, 2019; Keshavarzi & Haque, 2013; Rothman & Coyle, 2018; Mohamed, 1986; Abu-Raiya, 2014). Within the frameworks discussed by Keshavarzi & Haque (2013) and Rothman and Coyle (2018) human nature, *fitrah*, is seen as innately good. However, weakness in the *nafs*, due to greater influence of *al*-*nafs al-ammara*, leads to committing sin and thus it is interpreted to be the result of spiritual and mental weakness. The *‘aql* and *qalb* are important in modulating the influence of *al-nafs al-ammara* (Saniotis, 2018; Rothman & Coyle, 2018), while *al-nafs al-mutmaenna* is said to reflect the distinct Islamic framework in relation to self-actualisation and incorporating a spiritual dimension.

Through the expansion of the theories of Hippocrates and Galen by Muslim scholars, there was a shift from a belief in supernatural entities causing illness to an understanding based on a more scientific, rational basis of inquiry and investigation (Ammar, 1984; Dols, 1992; Ullmann, 1978; Watt, 1972). While magic was forbidden, lay beliefs continued to employ concepts such as “wise women”, “folk medicine”, and concepts of djinn possession and divine punishment (Dols, 1992). It is important to note that djinn do feature within pre-Islamic Arabic mythos and in Islamic theology – for example, Surah 72 within the Qur’an explicitly refers to djinn. That said, scholars and medical practitioners during the Islamic Golden Age, such as Ibn Sina, Abu Zayd al-Balkhi (850-934), al-‘Ibadi, al-Razi, and Ibn ‘Imran (d 903) rejected explanations based solely on folk belief and interpreted different manifestations of distress according to the humoural theory and the role of balance (Carnevali & Masillo, 2008; Porter, 1999; Dols, 1992; Skinner, 2010; Ullmann, 1978; Pormann & Savage-Smith, 2007; Awaad et al., 2019; Perez et al., 2012). Abu Sa’id Ibn Bakhtishu (940-1058) noted that, due to the interaction between the psychic (mental) and the physical, where a somatic condition causes a psychological effect, or, as they would have conceptualised it at the time, damage to the soul (*al-nafs*), treatment must be tailored to both – thus showcasing the consideration towards holistic health (Dols, 1984, 1992; Rahman, 1989; Ullmann, 1978). Ibn ‘Imran built on the work of Galen by advocating for the use of clinical observation and presentation before diagnosis, as well as establishing the patient’s temperament before the onset of illness (Omrani et al., 2012). These examples showcase how the discourse of mental illness was contemporaneously medicalised, in contrast to Medieval Europe where discourse of mental illness as result of demons, spirits, spiritual distress, and sin dominated even though knowledge about the Galenic and Hippocratic approaches to health existed (Pormann & Savage-Smith, 2007; Porter, 1999; Wallis, 2010).

Perhaps the most discussed mental health condition of the time was melancholia. Ishaq Ibn ‘Imran said that melancholia was a state of sadness which occurred due to excess black bile *(al-miras al-sawda’)* and a loss of *‘aql* (Ullmann, 1978). It was theorised that due to excess black bile, light (*‘aql*) was diminished, leading to feelings of powerlessness, dejection, isolation, and sadness (*al-huzn*) (Deuraseh & Talib, 2005; Saniotis, 2018). Al-Balkhi categorised *al-huzn* into three types: 1) everyday sadness; 2) sadness resulting from innate or pre-natal factors which is triggered by trauma or distress; and 3) sadness resulting from external events, such as immoderate eating, neglect of cleanliness of the body, or external disruption of the six non-naturals (Ullmann, 1978; Deuraseh & Talib, 2005). These categorisations have been described as analogous to current models of depression – that of 1) normal reaction to everyday life struggles, 2) endogeneous depression triggered by a specific instance as in line with the diathesis-stress model (Harrington & Clark, 1998; Colodro-Conde et al., 2018), and 3) exogenous/reactive depression (Haque, 2004; Ginter, Roysircar, & Gerstein, 2019; Saniotis, 2018). This demonstrates that the “nature-nurture” debate in mental ill-health was considered even during the Islamic Golden Age. Al-Balkhi was also the first to discuss the interconnectivity between physical and mental wellbeing by linking illness with the *nafs* to the development of physical ailments (Awaad et al., 2019; Deuraseh & Talib, 2005). Not only did he postulate this early approach to holistic health in his treatise *Masalih al-Abdan wa al-Anfus* (“Sustenance of the Body and Soul”), he also developed approaches that we would now view as cognitive and talking therapy. Indeed, he instructed individuals to keep helpful cognitions at hand during times of distress; employed persuasive talking, preaching, and advising; differentiated between normal and extreme emotional responses to situations; and studied the development of coping mechanisms for anger, fear, sadness, and obsessions (Awaad et al., 2019; Badri, 2013; Deuraseh & Talib, 2005). Badri (2013) draws an analogy between this framework of connecting cognitions and pathological behaviours to contemporary cognitive behavioural therapy, which uses cognitive restructuring and behavioural training. Al-Balkhi was also notable for distinguishing between neuroses and psychosis, classifying neuroses into four categories: fear and anxiety (*al-khawf wa al-faza’*), anger and aggression *(al-ghadab*), sadness and depression (*al-huzn wa al-jaza’*), and obsessions (*wasawes al-sadr*) (Awaad & Ali, 2015; Haque, 2004; Badri, 2013). Awaad and Ali (2015) argue that many of al-Balkhi’s categorisations of these conditions, which were based on symptomatic presentation, echo current diagnostic criteria in the DSM-5, such as that of obsessive compulsive disorder. However, they point out that differences in conceptualisation and terminology may result in an element of “presentism” in this analogy, referring to the act of applying present-day attitudes and standards to interpreting historical events. Badri (2013) suggests that al-Balkhi also was aware of the role of environmental influences on mental health, discussing the importance of public health factors, such as environment, pure water, clean air, housing, nutrition, and exercise. Al-Balkhi’s assertion that if the body becomes ill, then the soul is also afflicted (which, in turn, further affects the body) speaks to the integration of the holistic model of health and to the interplay between psychological and physical wellbeing (Deuraseh & Talib, 2005).

Abu Bakr al-Razi, considered to be one of the first practitioners of psychotherapy (*ilaj-al-nafsani*), is known for his many works on melancholia and madness (Ammar, 1984). He believed that sadness emerged due to an attachment to perishable things, or having lost something that one had possessed, which affected the balance between body and soul (Ammar, 1984). His treatise “On Spiritual Medicine” (*Kitab al-Tibb al-Ruhani*) discussed the importance of pure knowledge and the avoidance of “afflictions of the soul” (*‘awarid al-nafs*), which lead to impaired mental states (Pormann & Savage-Smith, 2007).

Ibn Sina, in “The book of the Cure” (*Kitab al-Shifa*), discussed using philosophy as a cure – with the reasoning that if mental illness results in a loss of reason, then philosophy could be used to combat ignorance, and ultimately mental illness (Pormann & Savage-Smith, 2007). His encyclopedia of medicine, *al-Qanun fi al-Tibb*, includes various descriptions of mental disorders, such as insomnia, amnesia, mania, hydrophobia, melancholia, etc. (Browne, 1921).

whilst melancholia was the most common mental health disorder described, many other conditions, such as mania and epilepsy, were said to be variants of melancholia, understood as burnt yellow bile interacting with excess black bile (Dols, 1992). Al-Razi believed that the symptoms of melancholia varied depending on where the excess black bile arose in the body: i.e., if it was in the brain, there would be mental confusion and delirium; if it arose from the whole body, there would be leanness and a flushed complexion (Dols, 1992). Ibn ‘Imran furthered this view by stating that madness could emerge from melancholia if there was severe depression due to a loss or separation. He differentiated melancholia into three types: 1) a cerebral type originating from a) burnt yellow bile and resulting in fever and sudden movements, foolish acts, and hallucinations, or b) bestial delusion caused by corrupt black bile; 2) black bile and burnt humours from the lower body rising to the brain; and 3) black bile in the body causing toxic vapours to affect the heart and brain (Dols, 1992: 72). Ibn ‘Imran argued that all these forms of melancholia involve fear, sadness, delusions, and hallucinations. In his *Qanun,* Ibn Sina refined the psychogenic theories of melancholia with the humoural theory and said that each humour leads to different susceptibilities to various mental disturbances: for example, black bile leads to anxiety, obsession, and melancholia; yellow bile results in irritation, inflammation, and delusions of fire; red bile leads to mental confusion, fevers, and epidemics; and phlegm leads to depression, sleepiness, obsessiveness, and delusions of being animals (Dols, 1992; Gorini, 2008). In order to care and treat those suffering from these conditions, institutions were set up to house and treat and care for those unwell.

## The development of hospitals

During the Islamic Golden Age, hospitals (*bimaristans*) were set up and funded by endowments from the state, *awqaf*, with male and female physicians from a variety of faiths and backgrounds (Ammar, 1984; Awaad et al., 2019; Mestiri, 2006; Gorini, 2008; Hamarneh, 1983; Pormann & Savage-Smith, 2007). There were places for the treatment and confinement of those with different illnesses. There are several instances within Islamic religious texts which are stated to have influenced public health approaches to care for the sick and unwell. For example, the Qur’an (4:5) states

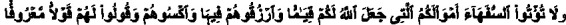

*“Do not give to those of weak of understanding your property which God assigned you to manage: but provide them from it, and clothe them, and speak kind and just words to them”.*

Additionally, we also see in the Qur’an (48:17), in reference to obligations upon the sick and infirm,

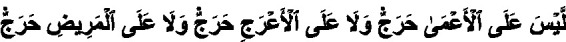

*“There is no blame for the blind, nor is there blame for the lame, nor is there blame for the sick”*

In the Hadith, in Sahih Bukhari (71:582) it is stated “There is no disease that Allah has created, except that He also has created its treatment.”

Inherent in the Hadith and *ayat* is a sense of absolving of responsibility of those that are sick (i.e., they are without blame and their illness is not a punishment). However, there is a responsibility on part of those with “property” to care for the unwell and to treat them, given that there is treatment for all conditions, as guaranteed by Allah. From this, one can see the religious justification for the development of institutions aiming explicitly to care for the “weak-minded”, based on the religious responsibility to care for the infirm. Interestingly, however, the management of these institutions, the *bimaristans*, was done in a secular way, despite being religiously influenced (Dols, 1984; Hamarneh, 1983; Pormann & Savage-Smith, 2007).

The Umayyad caliph, al-Walid (reigned 705-715), was said to have built a precursor to the modern hospital, by building an institution for the care of lepers and the blind (Hamarneh, 1983; Khan, 1986; Rahman, 1989). The first *bimaristan* to be established in the Medieval Muslim world was set up by Jibril Ibn Bakhtishu (d. 801) under the patronage of Caliph Harun al-Rashid (who reigned 786-809) (al-Issa, 2000; Dols, 1984; Hamarneh, 1983; Mestiri, 2006; Rahman, 1989; Watt, 1972). Through increased dissemination of Galenic works in Arabic, and an increased number of Muslim physicians, facilities for medical education were set up and often attached to hospitals (Dols, 1984). This link between highly reputable hospitals and medical education in its halls did not occur in Europe until the 16th century (Dols, 1984; Perez et al., 2012). It became customary within the *bimaristans* to develop admission records of patients and case reports, which were subsequently used in the training of medical practitioners (Hamarneh, 1983; Pormann & Savage-Smith, 2007). Hamarneh (1983) also explains that, in contrast to contemporaneous Europe where only monks and clergy were able to attend formal schooling, education (including medical education) was theoretically accessible to all strata within Muslim society. This would explain why, through the accommodation of the *dhimmis* (non-Muslims living under the protection of Muslim law), many Christian and Jewish doctors worked alongside Muslims as staff in the *bimaristans* (Dols, 1984; Hamarneh, 1983). Hospitals were built with the aim of serving the poor and marginalised in the towns and had generous endowments from the caliphs, such that the cost was often minimal or free to those admitted (Dols, 1984; Hamarneh, 1983; Rahman, 1989). The employment of the *dhimmis* is evidence that the hospitals operated as secular spaces and that they treated and employed people regardless of religious affiliation, reflecting a commitment to equity and justice (Hamarneh, 1983). Indeed, several generations of the Bakhtishu family, Nestorian Christians, were personal physicians of the caliphs (Mestiri, 2006; Pormann & Savage-Smith, 2007). These medical centres, which employed treatments using the scientific modalities of the day, stood in contrast to contemporaneous centres in Europe, which were predominantly religious institutions and largely seen as hospices for pilgrims, the poor, and the disabled, and which were run by religious institutions and monks (Awaad et al., 2019; Perez et al., 2012; Wallis, 2010). The religious elements were so infused within these European centres that beds were arranged so that the sick could see the altar when mass was celebrated (Awaad et al., 2019; Perez et al., 2012; Wallis, 2010). Ammar (1984) notes the secular nature of hospitals in the Islamic world, through the neutrality of medicine and science which essentially “*acted as common ground across races and religions and did not appear in Europe until much later*” (Ammar, 1984, p. 53).

The most famous hospitals in the Islamic world during this period were the ‘Adudi, Nuri, and Mansuri hospitals, named after their patrons. The ‘Adudi was built in Baghdad in 982 and had 24-28 doctors on staff, along with lecture hall facilities, a library, a prayer room, and a pharmacy (Hamarneh, 1983; Mestiri, 2006; Rahman, 1989). The Nuri was built in Damascus in 1146 (Hamarneh, 1983; Pormann & Savage-Smith, 2007). Doctors conducted rounds and had charts of patients (Rahman, 1989). The hospital had study and lecture rooms, and patients had special garments to wear, along with food being provided for them during their stay (Rahman, 1989). It had a good reputation for patient comfort (Hamarneh, 1983). The Mansuri was built in Cairo in 1284 under the patronage of the Caliph al-Mansur Qalawun, who was inspired by the Nuri hospital (Rahman, 1989). The Mansuri had separate wards for men and women and patient stay was not limited as it could accommodate 8000 people (Dols, 1992; Rahman, 1989; Watt, 1972). In the spirit of other hospitals at the time, the Mansuri was described as:

“[a] place of medical treatment for Muslim patients, male or female, rich and poor, from Cairo and the countryside of Egypt. Both residents and nonresidents from other countries, no matter what their race, religion, and so on [shall be treated here] for their full ailments, big or small, similar or different, whether the disease are perceptible [that is, are physical] or whether they are mental disturbances, because the preservation of mental order is one of the basic aims of the Shari’a. The foremost in [in law] is to be paid to those who have suffered loss of mind and hence loss of honour…. The hospital shall keep all patients, men and women, for treatment until they are completely recovered. All costs are to be borne by the hospital whether the people come from afar or near, whether they are residents or foreigners, strong or weak, low or high, rich or poor, the employed and the employers [that is, of all social classes], blind or sighted, famed or obscure, learned or illiterate. There are no conditions of consideration and payment; none is objected to or even indirectly hinted at from nonpayment. The entire service is through the magnificence of God, the generous one” (Rahman, 1989: 70)

The Mansuri, similar to the other great hospitals of the time, had lecture rooms, a library, as well as a chapel and a mosque (Hamarneh, 1983). While hospitals were seen in Egypt, Syria, and Iraq, there is no evidence to-date of similar institutions in Andalusia until the 14th century (Hamarneh, 1983; Mestiri, 2006; Perez et al., 2012).

What is important to note is that wherever these hospitals were built, they were the first efforts in public mental health, and inclusion and care for the socially marginalised and mentally ill.

## The treatment of the mentally ill in hospitals

Dols (1992) suggests that treatment wards for the mentally unwell in hospitals likely existed since the first hospitals emerged in the Medieval Islamic world. Dols (1992) mentions that, due to the stigma surrounding mental illness, it was likely only in serious cases that family members took their loved ones to the public hospitals for treatment. This would be especially true for women, who, given cultural factors, were not usually involved in public life. Nevertheless, Dols (1992) argues that the fact that there were female attendants and female-specific wards meant that there were serious enough cases that families allowed their female members to be treated at these hospitals, potentially under the care of a male physician. Additionally, Rahman (1989) notes that although treatment by a member of the same sex was preferable, gender-segregation was relaxed in the case of medical care, so men could treat women and vice versa. Family permission was an important consideration in treatment and it was usually up to the head of the household to determine whether they agreed with the treatment suggested by the physician (Awaad et al., 2019; Dols, 1984). Medicalised approaches to therapies employed in the *bimaristans* included treatments such as bloodletting, opium, aromatherapy, music therapy, *hijama* (cupping), and talk therapy, which shows that there was an evidence base for a plurality of treatment modalities, including those which may be seen as culturally contentious (e.g., music therapy through musicians playing the oud and nay, and talking therapies) (Ammar, 1984; Awaad et al., 2019; Pormann & Savage-Smith, 2007; Saniotis, 2018; Perez et al., 2012; Gorini, 2008). The fact that music therapy was used is particularly interesting. Otterbeck and Ackfeldt (2012) note that despite music having a role across many Muslim cultures, there are theological debates regarding its permissibility (i.e., Can it be performed with lyrics? With instruments?), given its ability to evoke strong emotions. This could, according to some conservative scholars, be seen as detracting from focusing on God and elucidating the base instincts of *al-nafs al-ammara* (Otterbeck & Ackfeldt, 2012).

The foremost therapy employed during the time, however, was of diet and regimen, followed by drug therapy, bloodletting, purges, emetics, cauterization, surgery, and, in severe cases, chaining (Awaad et al., 2019; Pormann & Savage-Smith, 2007; Carnevali & Masillo, 2008). With the belief that black bile caused a loss of moisture, it was thought that things which would calm the patient and bring back moisture would restore their mental functioning. Thus, therapies included keeping the patient in an area with moist air scented with herbs, giving them oils and pleasant scents, bathing them in lukewarm water, massaging their limbs, cupping (*hijama*), using compresses, and giving them a chamomile and poppy soporific to induce sleep (Dols, 1992; Awaad et al., 2019; Perez et al., 2012; Gorini, 2008). Additional therapies included shaving the head and poulticing the head with a mixture of mandrake and poppy seeds along with different types of oil to induce sleep and quell rage (Dols, 1992; Awaad et al., 2019; Gorini, 2008). Opium and cannabis were also used as soporifics, to calm the patient down (Dols, 1992; Gorini, 2008). Contemporaneous forms of medical treatment, such as ointments, herbs, pills, and liquids, were also used as treatment (Awaad et al., 2019). Present-day scholarship on the treatment of the mentally ill during this time often focuses on these treatments and may tend to “sanitise” accounts of mental health treatment. Thus, it is worth noting that chaining and confining of the mentally ill in these institutional spaces did occur, although there are debates as to its frequency and whether it was limited to the most severely unwell (Dols, 1992; Shoshan, 2003; Pormann & Savage-Smith, 2007). Nevertheless, as per their names as places of healing (*bayt al-shifa*) (Hamarneh, 1983), the focus was on convalescence and getting to a state of wellness and holistic health. This was conveyed through architectural design, with fountains and shrubbery placed in courtyards to evoke calmness and tranquility (Awaad et al., 2019; Carnevali & Masillo, 2008; Gorini, 2008), in addition to the therapeutic modalities described above. In line with this, it has been argued that patients were only chained “*until their reason is restored to them*” (Dols, 1992: 119) and as a therapeutic rather than punitive measure. Indeed, patients were regularly fed and monitored and those who were less severe were held in large rooms with ample sunlight (Carnevali & Masillo, 2008; Gorini, 2008).

Interestingly, there was an importance ascribed to the role of prayer (and ritualistic prayer) in affecting spiritual, psychological, physiological, and moral health, through listening to religious poetry and verses from the Qur’an. Yet, what is striking is that prayer was not seen as the sole or primary cure, as it often tends to be amongst contemporary Muslim populations, but was used in supplement with other conventional treatments (Ammar, 1984; Mestiri, 2006; Carnevali & Masillo, 2008; Hamarneh, 1983; Pormann & Savage-Smith, 2007).

## Conclusion

Having established how health and illness were viewed in the Islamic Golden Age, this article examined treatment facilities and methods for those afflicted by what would currently be conceptualised as mental disorders. Treatises by scholars in the Islamic Golden Age, such as those by Ibn Sina and al-Razi, built upon Galenic and Hippocratic theories of medicine to further their knowledge and understanding of mental illness. They would shift the discourse of mental ill-health amongst practitioners to employ a contemporaneous bio-medical paradigm – echoing the dominant model of mental illness today as an interaction between biological factors and psycho-socio-environmental stressors. This ran in stark contrast to the lay discourse of mental ill-health being due to magic, evil eye, and spirits/supernatural possession, which continues today amongst many Muslim communities.

During the Islamic Golden Age, public health approaches to mental health were evident through the designation of specialist wards for the mentally ill in hospitals funded by the *awqaf*, with special attention being paid to comfort and alleviation of discomfort and despair. Although Shoshan (2003) and Pormann and Savage-Smith (2007) suggest that Medieval Muslim hospitals operated more like contemporary hospices, we nevertheless see that Muslim physicians had innovative ideas about the treatment of the mentally ill, even though the physicians themselves may not have all agreed on the exact pathophysiology of the development of these disorders. However, what this demonstrates is that, during the Islamic Golden Age, mental disorders were seen as phenomena that existed, requiring clinical assessment and treatment, and categorised and assessed systematically by employing rational judgements and observation rather than cultural beliefs based on supernatural causes. The plurality of treatment modalities used, justified Islamically within the concept of *shifa* (healing), is striking given contemporary debates regarding whether these are *haraam* (forbidden) in Islam (Harris, 2002; Otterbeck & Ackfeldt, 2012). Additionally, whilst notions of stigma may have existed, those suffering from mental health conditions were not necessarily ostracised.

What is remarkable is that there is a historical record of mental illness viewed within the Islamic tradition employing contemporaneous medical discourse, i.e., that of the humours, whilst also advocating for various humane treatments and therapies in institutionalised care settings. This runs counter to the prevalent discourse of Muslim mental health, as seen through an Orientalist lens, in which Muslims are said to believe that supernatural spirits cause mental illness and treatment is left to fatalistic thinking. Whilst “folk models” of mental illness do exist in lay discourse amongst contemporary Muslim communities, which echo to some extant European frameworks of healing and illness in the Middle Ages, the contemporary movement of Islamic psychology being developed by Muslim mental health scholars may be seen as a reclamation of the Islamic teachings and frameworks in mental health during the Golden Age (Skinner, 2010, Rothman & Coyle, 2018). This movement could also be read as a response to the increasing demand to outline distinct “Islamic” approaches to mental health. This may explain findings by Hussain and Cochrane (2002) and Razali and Tahir (2017), which indicate that Muslim communities may contest local cultural attitudes and favour approaches framed within a religious discourse, even if only nominally. This is where, from a clinical perspective, it becomes crucial to understand the importance of religious affinity and religious practise. As with adherents to world religions more broadly, there is a sense of community, solidarity, and authenticity that membership of a superordinate body, such as a religious affiliation, brings (Kibria, 2008; Ryan, 2014; Valentine & Sporton, 2009; Ysseldyk, Matheson and Anisman, 2010).

This article has shown that, in the Islamic tradition, a specific focus on holistic health and duty of care to the sick informed the development of discourses, moved beyond cultural models, to more investigatory, systematic, and empirical methods. As such, the Islamic Golden Age heralded the development of public health approaches to mental health ensconced within the bio-medical paradigm of the day. It may be important for clinicians who work with Muslim patients with strongly held cultural beliefs to note that bio-medical therapies and treatments do have a foundation within the Islamic tradition and have a precedence of being used via the Islamic concept of *shifa*. It is important to understand the approaches used by Islamic scholars towards mental ill-health during the Islamic Golden Age as a means of understanding the foundation of Islamic psychology, as the movement claims its lineage. This analysis has its own intrinsic significance as a means of understanding frameworks and approaches to mental health that Muslims had used, in relation to contemporary approaches to mental health and illness. Of note is how scholars and practitioners used contemporaneous medical knowledge alongside religious influences to develop a distinct branch of Islamic medicine and understanding of mental health. The increased proliferation of, and demand and market for, “Muslim mental health” would suggest that whilst efforts to address mental health within a religio-cultural framework for Muslims is laudable, further research is necessary (Younis, 2019). Does “Islamic psychology” merely adopt the approaches of scholar-physicians during the Golden Age as an “Islamic” “closed corpus”? Should practitioners work within contemporary paradigms of medical discourse adapted through the lens of religion? These are questions which require further development and investigation in our understanding and approaches to mental health amongst Muslim communities.
